# Quantifying protein digestion kinetics of feed ingredients using a modified in vitro incubation assay

**DOI:** 10.1093/jas/skaf190

**Published:** 2025-06-18

**Authors:** Shiyi Zhang, Leon de Jonge, Sonja de Vries, Walter J J Gerrits

**Affiliations:** Animal Nutrition Group, Wageningen University & Research, Wageningen, The Netherlands; State Key Laboratory of Animal Nutrition, College of Animal Science and Technology, China Agricultural University, Beijing, China; Animal Nutrition Group, Wageningen University & Research, Wageningen, The Netherlands; Animal Nutrition Group, Wageningen University & Research, Wageningen, The Netherlands; Animal Nutrition Group, Wageningen University & Research, Wageningen, The Netherlands

**Keywords:** amino acids, hydrolysis, peptides, solubilization

## Abstract

The kinetics of degradation of nutrients from diets or ingredients contributes to the nutritional value of feed ingredients. The aim of this study was to quantify the kinetics of nitrogen (N) solubilization and hydrolysis for feed ingredients varying in protein content and ileal protein digestibility by a modified 2-step enzymatic in vitro incubation assay. The amount of N subjected to in vitro incubation was standardized (approximately 0.48 g) among selected feed ingredients (rapeseed meal (RSM), fish meal, barley, peas, and zein). At the beginning of the stomach phase with pepsin, pH was set at 4 and then adjusted to 2 after 90 min, followed by a further 90 min of incubation. Subsequently, pH was adjusted to 6.8 and after 15 min, pancreatin and amyloglucosidase were added to simulate small intestinal digestion for 240 min. Total solubilized nitrogen (**TSN**) and low molecular weight nitrogen compounds (**LMWN**, <500 Da) were measured at several time points (0 to 435 min) during incubation. Flavourzyme (**FL**) or intestinal acetone powders from rat (**IAPR**) were added for 1 additional hour of incubation following the small intestine phase to simulate brush-border enzyme activity. Between 46% (RSM) and 73% (fish meal) of the TSN, and 39% (RSM) to 68% (fish meal) of the LMWN were seen during the stomach phase. Nitrogen solubilization in response to pH-change during the stomach phase was most pronounced in fish meal, having the highest rate of N solubilization during the first 10 min after adjusting pH from 4 to 2 (3.10%/min) and the highest N solubility (73%) and LMWN (68%) during the stomach phase. The kinetics of appearance of LMWN followed the pattern of N solubilization. During small intestinal phase, proteins in RSM and barley exhibited higher solubilization and hydrolysis yet achieved slightly lower solubility (76% for RSM and 77% for barley) and LMWN (73% for RSM and 70% for barley) compared with fish meal (86% for N solubility and 82% for LMWN) and peas (85% for N solubility and 72% for LMWN). The addition of FL or IAPR did not increase N solubilization or LMWN. Our modified in vitro assay allowed to quantify rate and extent of N solubilization and appearance of LMWN of feed ingredients varying in protein content and ileal digestibility, reflecting variation in protein characteristics, with potential effects on absorption kinetics in vivo.

## Teaser

Changing the pH during analysis of digestion kinetics in vitro from 4 to 2 during the stomach phase resulted in a substantial increase of nitrogen solubilization and hydrolysis for fish meal compared with other selected feed ingredients. The extent of nitrogen solubilization and hydrolysis remained consistent across the selected feed ingredients before and after the additional incubation with brush-border enzymes.

## Introduction

Total tract and ileal digestibility values are commonly used to predict nutritional values of feed ingredients (NRC, 2012; MAFIC, 2020; CVB, 2021). For amino acids, standardized ileal digestibility values refer to the extent of digestion at the end of the small intestine and do not reflect the kinetics of absorption in the small intestine. Synchronization of protein and starch digestion, and subsequent absorption, has the potential to reduce the extent of amino acid oxidation, thus impacting protein deposition in pigs (Van den Borne et al., 2007). The rate at which nutrients enter the bloodstream can impact their availability for post-absorptive metabolism (Batterham and Bayley, 1989; Boirie et al., 1997; Dangin et al., 2001). Proteins that are quickly digested, such as whey protein, lead to a more rapid postprandial appearance of amino acids and peptides in the blood, compared with slow-digested sources, like casein (Boirie et al., 1997; Chen et al., 2019). Soluble proteins are susceptible to hydrolysis by digestive enzymes in the gastrointestinal tract, facilitating rapid breakdown into smaller molecules. Moreover, flowing with the liquid phase of digesta, soluble—or solubilized proteins—are typically rapidly emptied (from 16 to 397 min) from the stomach, whereas insoluble proteins, flowing with the solid phase, are retained longer (from 54 to 612 min) (Montenegro, 2024). Consequently, soluble proteins arrive earlier in the small intestine than insoluble proteins, allowing them to undergo hydrolysis by pancreatic enzymes and absorption quicker (Schop et al., 2023; Roelofs et al., 2024). Therefore, in feed evaluation, measuring the kinetics of nutrient solubilization and break down is crucial for accurate predictions of nutrient absorption and the nutritional value of feed ingredients.

To assess the kinetics of protein solubilization and hydrolysis, in vitro assays are commonly employed (Wilfart et al., 2008; Chen et al., 2019; Schop, 2020; Sousa et al., 2020; Pälchen et al., 2021). These approaches were mostly adapted from stepwise enzymatic hydrolysis assays developed by Boisen and Fernandez (1995) and the INFOGEST protocol (Brodkorb et al., 2019), relying on the incubation of substrates with pepsin and pancreatic enzymes under standardized conditions. It is typically assumed that proteins solubilized during such in vitro incubation procedures reflect the fraction digested in vivo. Compared with the Boisen and Fernandez (1995) method, the INFOGEST protocol is more complex to perform. The Boisen method has been validated as a simple and reliable in vitro approach for assessing organic matter digestibility in pig feed ingredients, with results comparable to in vivo findings. Therefore, to assess protein solubilization and hydrolysis kinetics in pig feed ingredients in a straightforward and standardized manner, the Boisen method have been chosen.

These assays have been proven useful to assess protein digestion kinetics in vitro, but several caveats have been addressed. Firstly, relying on the quantification of solubilized proteins after incubation with pepsin and pancreatic enzymes to reflect digestibility, in vivo digestibility may be overestimated. Solubilized proteins are not per se absorbed by enterocytes, if not yet hydrolyzed into smaller peptides and amino acids. As proposed by Chen et al. (2019), quantification of the appearance of low molecular weight peptides and amino acids (**AA**) could be used to complement N-solubilization measurements to provide further insights into absorption kinetics. Conversely, as most in vitro assays do not include peptidase activities from several enzymes secreted by the small intestinal brush border—which are responsible for the degradation of poly- and oligopeptides into absorbable peptides and AA in vivo, the appearance of low molecular weight peptides and AAs may actually be underestimated compared with the in vivo situation. The inclusion of brush border enzyme activities in vitro assays may thus be crucial to accurately mimic protein digestion. This was illustrated by a study of Picariello et al. (2015), where the addition of brush border enzymes to an in vitro assay increased the degree of protein hydrolysis of milk products by approximately 16%. Lastly, gastric pH is not constant and depends on the diet characteristics (Merchant et al., 2011; Bornhorst et al., 2014). The effect of pH on protein solubility is considerable (Xia, 2018) and varies across proteins. Although, mimicking in vivo pH-fluctuations in the stomach over time is extremely complex and beyond scope, assessing the response in protein solubilization after a pH-change based on the gastric pH, can reveal relevant information about the protein characteristics and may contribute to a better understanding of variation in in vivo protein digestibility among feed ingredients.

The aim of the current study was to quantify the kinetics of nitrogen (N) solubilization and hydrolysis for feed ingredients varying in protein concentration and ileal digestibility, using a modified Boisen-based (1995) in vitro assay as described by Schop (2020). Five feed ingredients were selected to serve as model ingredients representing high (fish meal, rapeseed meal, zein) or low (barley, peas) protein concentrations varying in ileal digestibility. The assay described by Schop (2020) was adapted as follows: 1) standardizing the quantity of N incubated instead of sample weight to ensure levels of low molecular weight nitrogen compounds (**LMWN**) in the incubation liquid above the detection limit; 2) the introduction of a 2-phase pH step during the stomach phase to figure out the effect of pH change on protein solubilization and hydrolysis. Furthermore, the effect of adding brush border enzymes on protein hydrolysis kinetics was tested by adding a commercial peptidase originating from *Aspergillus oryzae* (Flavourzyme, FL) or brush border enzymes isolated from rat intestinal mucosa (intestinal acetone powders from rat, IAPR), on the completion of incubation with pancreatic enzymes.

## Materials and Methods

### Materials

Rapeseed meal (RSM), fish meal, peas, and barley were obtained from single batches of commercially available feed ingredients (Schop, 2020) and ground to pass a 1 mm sieve at 12000 rpm (Retsch ZM 200, Haan, North Rhine-Westphalia, Germany). Zein was obtained from Sigma-Aldrich Ltd. (St. Louis, MO, USA) and used as such.

Pepsin (2,000 FIP U/ g), pancreatin (4 × USP specifications, porcine pancreas grade VI), amyloglucosidase (from *Aspergillus Niger*, 120 U/mg), intestinal acetone powders from rat (IAPR, I1630), Flavourzyme (**FL**) (protease from *Aspergillus oryzae*, ≥500 U/g) and 5-sulfosalicylic acid (**SSA**)-dehydrate were obtained from Sigma-Aldrich Ltd. (St. Louis, MO, USA).

### In vitro nitrogen-degradation kinetics assay

Kinetics of N solubilization and hydrolysis of the 5 feed ingredients were measured in duplicate, using the method of Schop (2020) with modifications. Briefly, samples (0.48 ± 0.001 g of N) were weighed into 600 mL beakers mixed with preheated (39 °C) disodium phosphate buffer (250 mL, 0.1M, pH 6.0) and hydrochloric acid (HCl, 100 mL, 0.1M), and incubated in a water-bath at 39 °C under constant magnetic stirring (300 to 340 rpm). The pH was adjusted to 4 with 10M sodium hydroxide (NaOH) solution. To stabilize the incubation system, the suspension was incubated under continuous stirring without enzymes for 1 h. After 1h of incubation, 30 mL aliquot of the suspension was taken as time 0 sample. Then 10 mL of freshly prepared pepsin solution (0.025 g/mL) was added. After 90 min, pH was adjusted to 2 with 6M HCL for the second half of the incubation (90 to 180 min). A 30 mL aliquot of the suspension was taken from each beaker at time 10, 20, 30, 60, 90, 120, and 180 min for analyses of total solubilized N (**TSN**) and LMWN for the kinetics of N solubilization and hydrolysis. An extra 15 mL aliquot of the suspension were taken at time 100 min for checking the pH response of N solubilization after adjusting pH from 4 to 2.

Combined stomach and small intestine phases with no intermediate sampling during the stomach phase ran independently from the sampled stomach-only incubations, following the methodology outlined by Schop (2020). Following the stomach phase, 50 mL NaOH (0.6M) and 100 mL sodium phosphate buffer (0.2M, pH 6.8) were added. The suspension was adjusted to pH 6.8 with HCL (6M). To stabilize the incubation system, the suspension was incubated under continuous stirring without intestinal enzymes for 15 min, after which, a 30 mL aliquot of the suspension was taken as time 0 sample of small intestine phase. Then, the beakers were incubated in a water-bath at 39 °C for 240 min under continuous magnetic stirring (280 to 320 rpm). Freshly prepared pancreatin solution (10 mL, 0.1 g/mL, non-solubilized material was removed by centrifugation 4,000 × *g* for 15 min) and 27.5 mg of amyloglucosidase was added. A 30 mL aliquot of the suspension was taken from each beaker at time 0, 10, 20, 30, 60, 120, and 240 min under continuous stirring for determination of TSN and LMWN. An additional 20 mL aliquot of the suspension from each beaker was taken at the end of the incubation for incubation with FL or IAPR.

In the 20 mL aliquot of the suspension taken at the end of small intestine phase (240 min) 1.5 mL FL (Rovalino-Córdova et al., 2019) or freshly prepared IAPR (0.02 g/mL) (Vermeirssen et al., 2005) was added and incubated for another 1h at 39 °C under constant magnetic stirring (280 to 320 rpm). A blank was included in every incubation phase for correcting the TSN and LMWN originating from enzymes.

The suspension samples taken at consecutive points were centrifuged (4,000 × *g*, 15 min) to separate solid and liquid phases. Then, 10 mL of the supernatant was decanted for analysis of TSN. The other 5 mL of the supernatant was mixed with 5 mL SSA (16% w/v) to precipitate high molecular weight N (1:1 v/v) and then centrifuged (at 10,000 × *g*, 10 min). The N concentrations of feed ingredients and TSN and LMWN in the supernatants were analyzed using the Kjeldahl method (ISO 5983-2:2005; FOSS 8400 Analyzer unit and 8420 Sampler).

### Calculations and presentation of data

At each time point, N solubility and appearance of LMWN were calculated as follows:


$$\begin{array}{l} N\;solubility_{i}(\%)=(N_{solubilized,i}-N_{blankT})/\;N_{substrate}\times 100\% \end{array}$$

$$\begin{array}{l} LMWN_{i}(\%)=(N_{LMWN,i}-N_{blankL})/\;N_{substrate}\times 100\% \end{array}$$

Where N solubility_i_ and LMWN_i_ is the amount of total soluble or LMWN found in the incubation suspension at the *i*th time point relative to the total amount of N incubated (%), N_solubilized,i_ is the amount of solubilized N at the *i*th time point (g), N_blankT_ is the average of the amount of solubilized N found in the incubation suspension of blank samples during the stomach or small intestine phase without substrate (g) and thus represents the solubilized N originating from enzymes, N_substrate_ is the amount of N in the incubated sample N_LMWN,i_ is the amount of LMWN in the soluble N fraction at the *i*th time point (g), N_blankL_ is the average of the amount of LMWN found in the incubation suspension of blank samples during the stomach or small intestine phase without substrate (g) and thus represents the LMWN originating from enzymes. N_solubilized_ and N_LMWN_ were corrected for the N removed from the incubation with subsequent aliquots at preceding time points. The rates of N solubilization and appearance of LMWN (%/min) for the feed ingredients were estimated from slopes of single linear increase for each time interval:

- 0 to 90 min,
- 90 to 100 min (pH response 1),
- 100 to 180 min in stomach for N solubilization or 90 to 180 min for appearance of LMWN,
- 180 to 195 min (pH response 2),
- 195 to 215 min,
- 215 to 435 min.

One replicate sample of peas for initial N solubility during the stomach phase (0 min) and fish meal for final N solubility during the small intestine phase (435 min) were excluded due to measurement error.

## Results

As the experimental design does not allow statistical evaluation of observed differences, results are presented as a narrative description and any reference to differences between feed ingredients are indicative rather than statistically analyzed. During the incubations, zein exhibited distinct behavior compared with the other feed ingredients, notably displaying agglutination, which resulted in significant variability between replicates and difficulties in obtaining representative suspension samples (Table S1). Hence, this feed ingredient was excluded from further calculation of protein solubilization and hydrolysis kinetics. Briefly, N solubilization and appearance of LMWN of zein increased rapidly during the stomach phase. After 120 min incubation in the stomach phase, N solubility and appearance of LMWN exceeded those observed during small intestine phase.

### The kinetics of nitrogen solubilization

**Figure 1.**
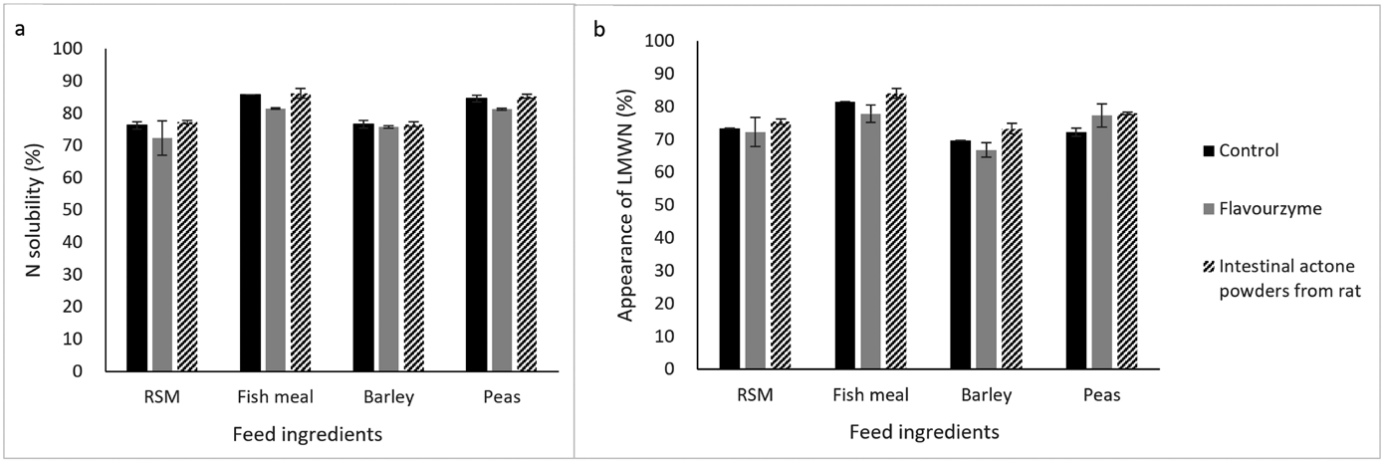
The effect of the addition of Flavourzyme or intestinal actone powders from rat on nitrogen (N) solubility (panel a) or appearance of low molecular weight nitrogen (LMWN) (panel b) during the subsequent 60 min incubation after a 3-step enzymatic in vitro incubation for rapeseed meal (RSM), fish meal, barley and peas. Error bars represent standard error of the mean, of 2 replicate observations, except for N-solubility of fish meal control where *n* = 1.

For all feed ingredients, the majority of N was solubilized during the stomach phase, ranging from 46% for RSM to 73% for fish meal (Table 1). The initial solubility of N at start of the stomach phase (pH 4) varied across feed ingredients, ranging from 20% for peas to 9% for RSM. The rate of N solubilization at pH 4 (0 to 90 min) increased in the order of fish meal (0.10), RSM (0.14), barley and peas (approximately 0.30/min). Particularly after adjusting to pH 2 (90 to 100 min of stomach phase), considerable N solubilization was observed and the variation in the rates of N solubilization among feed ingredients became more pronounced. fish meal displayed the greatest response in the rate of N solubilization upon a change in pH from 4 to 2 (3.10%/min), followed by peas (2.51%/min), RSM (1.23%/min), and barley (0.84%/min). In contrast to the stomach phase at pH 4, at pH 2 (100 to 180 min of stomach phase) fish meal (0.27%/min) and RSM (0.16%/min) showed higher rates of N solubilization compared with barley (0.06%/min) and peas (0.04%/min). At the end of the stomach phase, notable differences in the total amount of N solubilized were observed among feed ingredients, where fish meal and peas showed N solubility with over 68% while RSM and barley showed less than 56%. The increase of pH from 2 to 6.8 at the onset of the small intestine phase (180 to 195 min) led to continued N solubilization for all feed ingredients except barley. Negative rate of N solubilization was observed for barley (−0.29%/min), presumably explained by the lower N solubility at time 195 min than 180 min in one of the replicates. During small intestinal incubation, the addition of pancreatin facilitated the rate of N solubilization in RSM (0.54%/min) and barley (1.01%/min), but not in fish meal (−0.06%/min) and peas (−0.02%/min) in the initial 20 min (195 to 215 min). Subsequently, N solubilization reached a plateau and remained constant until the end (215 to 435 min). At the end of small intestine phase, fish meal and peas (approximately 85%) exhibited slightly higher N solubility than RSM and barley (approximately 75%). No effect of additional incubation, following the small intestine phase, with FL or IAPR for 1h on the final N solubility was observed, regardless of feed ingredient (Fig. 1).

**Table 1.** Nitrogen (N) concentration and kinetics of N solubilization for rapeseed meal (RSM), fish meal, barley, and peas during a 2-step enzymatic in vitro incubation^1^ conducted in duplicate (*n* = 2)

	RSM	fish meal	Barley	Peas	Pooled SEM
N content, g/kg as-is	55.5	114.8	16.2	32.9	
Stomach^2^					
Initial solubilization, 0 min, %	8.7	11.6	13.5	19.8^5^	0.1
Solubilization rate phase 1, 0 to 90 min, %/min	0.14	0.10	0.31	0.29	0.06
pH response, 90 to 100 min, %/min	1.23	3.10	0.84	2.51	0.04
Solubilization rate phase 2, 100 to 180 min, %/min	0.16	0.27	0.06	0.04	0.04
Endpoint solubilization, 180 min, %	46.2	73.0	55.2	68.2	1.2
Small intestine^3^					
pH response, 180 to 195 min, %/min	0.38	0.53	−0.29	0.86	0.66
Initial solubilization, 195 min, %	52.0	81.0	50.8	81.1	10.7
Solubilization rate phase 1, 195 to 215 min, %/min	0.54	−0.06	1.01	−0.02	0.41
Solubilization rate phase 2, 215 to 435 min, %/min	0.06	0.04^4^	0.03	0.02	0.01
Endpoint solubilization, 435 min, %	76.3	85.8^4^	76.6	84.5	2.2

^1^In vitro incubation with pepsin at pH 4 for 90 min and pH 2 for 90 min (stomach) followed by incubation with pancreatin at pH 6.8 for 240 min (small intestine).

^2^Initial solubilization, initial N solubility at the start of stomach phase (0 min); Solubilization rate phase 1, the rate of N solubilization between 0 and 90 min of stomach phase at pH 4; pH response, the increase of N solubilization due to the pH adjustment from pH 4 to 2 at 90 min (90 to 100 min); Solubilization rate phase 2, the rate of N solubilization between 100 and 180 min of stomach phase with at pH 2; Endpoint solubilization, final N solubility at the end of stomach phase (180 min).

^3^pH response, the increase of N solubilization due to the pH adjustment from 2 to 6.8 at 180 min between 180 and 195 min of small intestine phase without adding pancreatin and amyloglucosidase; Initial solubilization, initial N solubility at the start of small intestine phase (195 min); Solubilization rate phase 1, the rate of N solubilization between 195 and 215 min of small intestine phase at pH 6.8; Solubilization rate phase 2, the rate of N solubilization during 215 to 435 min of small intestine phase with pH 6.8; Endpoint solubilization, final N solubility at the end of small intestine phase (435 min). ^2,3^In each incubation period, solubilization rate was estimated as the slope of the linear model: N solubility_j_ = N solubility_i_ + solubilization rate*t, where N solubility_j_ (%) is the N solubility at 90, 100, 180, 195, 215 and 435 min, N solubility_i_ (%) is the N solubility at 1 previous sampling time point (0, 90, 100, 180, 195 and 215 min), and t (min) is the incubation time of the 6 phases.

^4^One replicate was removed as outlier. Pooled SEM of solubilization rate phase 2 and endpoint solubilization of small intestine phase were based on the comparison of RSM, barley, and pea samples only.

^5^One replicate was removed as outlier. Pooled SEM of initial solubilization of stomach phase were based on the comparison of RSM, fish meal and barley samples only.

### The kinetics of appearance of low molecular weight nitrogen

In general, the kinetics of appearance of LMWN reflected kinetics of N solubilization (Tables 1 and 2). The initial LMWN at start of the stomach phase was higher for peas (17%) compared with the other feed ingredients (7% to 12%). The rate of appearance of LMWN at pH 4 (0 to 90 min) increased in the order fish meal (0.06), RSM (0.14), peas (0.20), and barley (0.27%/min), in accordance with patterns of N solubilization. Similar as observed for N solubilization, differences in the rates of appearance of LMWN among feed ingredients became more pronounced when pH was changed from 4 to 2 (90 to 180 min). For fish meal, the rate of appearance of LMWN increased nearly 10 times compared with the 0 to 90 min of the stomach phase (0.57%/min vs. 0.06%/min), whereas for barley, the rate was decreased (0.27%/min vs. 0.17%/min). Consequently, fish meal had highest LMWN at the end of stomach phase (68%) compared with other feed ingredients (39% to 62%). The ranking of feed ingredients was consistent for both LMWN and N solubilization, though LMWN were approximately 6% lower than N solubility at the end of stomach phase. Changing pH from 2 to 6.8 at the onset of the small intestine phase (180 to 195 min) resulted in continued appearance of LMWN for RSM, fish meal and peas, whereas virtually no more LMWN for barley. During small intestine phase with the addition of enzymes, LMWN continuously appeared within the initial 20 min for barley (0.71%/min) and peas (0.16%/min). The addition of enzymes did not facilitate the rate of appearance of LMWN for RSM (0.01%/min) and fish meal (−0.10%/min). Similar to the observed kinetics of N solubilization, appearance of LMWN reached a plateau during the 214 to 435 min of small intestine phase. The final extent of LMWN was however considerably lower (3% to 12%) than that of N solubilization. There was no effect of additional incubation, following the small intestine phase, with FL or IAPR on the final LMWN, for all ingredients (Fig. 1).

**Table 2.** Kinetics of low molecular weight nitrogen (LMWN) appearance for rapeseed meal (RSM), fish meal, barley, and peas during a 2-step enzymatic in vitro incubation conducted in duplicate (*n* = 2)^1^

	RSM	fish meal	Barley	Peas	Pooled SEM
Stomach^2^					
Initial LMWN, 0 min, %	8.6	11.3	11.8	17.4	2.0
Appearance of LMWN rate phase 1, 0 to 90 min, %/min	0.14	0.06	0.27	0.20	0.03
Appearance of LMWN rate phase 2, 90 to 180 min, %/min	0.19	0.57	0.17	0.30	0.02
Endpoint LMWN, 180 min, %	38.8	68.1	51.5	62.4	3.3
Small intestine^3^					
pH response, 180 to 195 min, %/min	1.35	0.67	−0.03	0.35	0.29
Initial LMWN, 195 min, %	59.0	78.1	51.0	67.6	6.1
Appearance of LMWN rate phase 1, 195 to 215 min, %/min	0.01	−0.10	0.71	0.16	0.27
Appearance of LMWN rate phase 2, 215 to 435 min, %/min	0.06	0.03^4^	0.02	0.01	0.01
Endpoint LMWN, 435 min, %	73.4	81.5^4^	69.7	72.3	1.4

^1^In vitro incubation with pepsin at pH 4 for 90 min and pH 2 for 90 min (stomach) followed by incubation with pancreatin at pH 6.8 for 240 min (small intestine).

^2^Initial appearance of LMWN, initial appearance of LMWN at the start of stomach phase (0 min); appearance of LMWN rate phase 1, the rate of appearance of LMWN between 0 and 90 min of stomach phase at pH 4; appearance of LMWN rate phase 2, the rate of appearance of LMWN between 90 and 180 min of stomach phase with at pH 2; Endpoint appearance of LMWN, final appearance of LMWN at the end of stomach phase (180 min).

^3^pH response, the increase of appearance of LMWN due to the pH adjustment from 2 to 6.8 at 180 min between 180 and 195 min of small intestine phase without adding pancreatin and amyloglucosidase; Initial appearance of LMWN, initial appearance of LMWN at the start of small intestine phase (195 min); appearance of LMWN rate phase 1, the rate of appearance of LMWN between 195 and 215 min of small intestine phase at pH 6.8; appearance of LMWN rate phase 2, the rate of appearance of LMWN during 215 to 435 min of small intestine phase with pH 6.8; Endpoint appearance of LMWN, final appearance of LMWN at the end of small intestine phase (435 min). ^2,3^In each incubation period, appearance of LMWN rate was estimated as the slope of the linear model: appearance of LMWN_j_ = appearance of LMWN_i_ + appearance of LMWN rate*t, where appearance of LMWN _j_ (%) is the appearance of LMWN at 90, 180, 195, 215 and 435 min, appearance of LMWN _i_ (%) is the appearance of LMWN at 1 previous sampling time point (0, 90, 180, 195 and 215 min), and t (min) is the incubation time of the 5 phases.

^4^One replicate was removed as outlier. Pooled SEM of appearance of LMWN rate phase 2 and endpoint appearance of LMWN of small intestine phase were based on the comparison of RSM, barley, and pea samples only.

## Discussion

The aim of current study was to quantify the kinetics of N solubilization and appearance of LMWN of feed ingredients differing in protein content and ileal digestibility, using a modified Boisen-based in vitro model. Overall, the majority of N was solubilized and hydrolyzed during the stomach phase, with the highest solubility and LMWN observed for fish meal (73% and 68%) and the lowest for RSM (46% and 39%). The appearance of LMWN exhibited a similar trend as the kinetics of N solubilization. The measurement of N solubilization during the stomach phase is relevant, because soluble proteins, are swiftly emptied from the stomach and thus can be digested and absorbed more rapidly compared with insoluble proteins (Schop et al., 2023).

### Standardizing the incubated nitrogen amount alleviated the quantification limitations of nitrogen concentrations in liquid samples

Commonly used in vitro assays rely on the quantification of protein digestibility by 1) the solid residues obtained after centrifugation of a series of samples incubated for progressively increasing time intervals (Wilfart et al., 2008; Chen et al., 2019); or in 2) repeated samples of the whole suspension taken at various time points while stirring during incubation of feed ingredients (Schop, 2020), with fixed pH during the stomach phase. In the current study, the latter strategy of taking aliquots of the suspension at several time points was employed. Standardizing the amount of N incubated, which is one of the modifications to the assay presented by Schop (2020), alleviated the quantification limitations of LMWN concentrations in liquid samples, particularly for feed ingredients with low protein content. This ensured consistent findings of N solubilization and appearance of LMWN, even for barley that has a low protein content (101 g/kg as-is). We further validated our assay by comparing the extent of protein solubilization for the selected feed ingredients in the small intestinal phase, which aligned well with previous studies (Chen et al., 2019; Schop et al., 2020) despite using different batches of enzymes (Sigma/Merck), thereby confirming the robustness of our approach. Additionally, a N recovery test was conducted prior to the experiment using the same feed ingredients to validate the strategy of taking aliquots of the suspension. Both liquid and solid residues were collected at 20 min after the start and at the end of the stomach and small intestine phase. Nitrogen recovery was on average 93% in both stomach and small intestine phases, demonstrating that the method achieves acceptable N recovery levels.

### The decrease of pH from 4 to 2 during the stomach phase substantially increased N solubilization and hydrolysis

The greater increase in N solubility and LMWN during the stomach phase compared with the small intestine phase indicates that the pH-dependent protein solubilization seems to be more of limiting step than subsequent hydrolysis by pancreas enzymes in this in vitro model. This observation is consistent with the findings of Mulet-Cabero et al. (2020). Furthermore, it also aligns with a previous study where setting the pH to 2 during pepsin incubation resulted in protein solubilization exceeding 80% by the end of the incubation for wheat, barley, and soybean meal, with negligible hydrolysis occurring afterwards with pancreatin (Wilfart et al., 2008). In addition, the observation that most solubilization occurred within 10 min of decreasing the pH from 4 to 2 suggests that pH-dependent structural changes in proteins play a more significant role in limiting protein solubilization than enzymatic hydrolysis. At low pH, particularly when changing pH from 4 to 2 during the stomach phase, proteins unfold due to intramolecular charge repulsion (Fink et al., 1994). This unfolding likely enhances the accessibility of protein cleavage sites for enzymatic hydrolysis. Furthermore, pepsin, which has an optimal activity at pH 2, profoundly facilitated protein solubilization and hydrolysis under these conditions (Piper and Fenton, 1965). In the current study, the pH adjustment during the stomach phase resulted in approximately 10% to 20% higher N hydrolysis compared with previous in vitro studies based on the Boisen assay with a fixed pH of 3.5 on RSM (Chen et al., 2019) or the same samples of RSM, fish meal, barley and peas (Schop, 2020). Indeed, our results of N solubilization for barley are comparable to the 54% found by Wilfart et al. (2008) when employing a pH of 2 in the stomach phase. In addition, the kinetics of absorption observed in vivo, as measured through blood sampling from the portal vein of catheterized pigs, are significantly faster than appearance of LMWN in in vitro systems (Schop et al., 2023). Enhancing the kinetics of N hydrolysis by adjusting the pH during the stomach phase may help increase the maximum in vitro rate. The current results underline the importance of pH adjustment during the stomach phase in facilitating N solubilization and appearance of LMWN.

### Precipitation of proteins

The original Boisen method relies on the quantification of solubilized proteins after incubation with pepsin and pancreatic enzymes to reflect digestibility. However, this approach may overestimate in vitro digestibility as solubilized proteins are not per se absorbed by enterocytes, if not yet hydrolyzed into smaller peptides and AAs. Hence, we also studied the appearance of LMWN in the suspensions. To this end, we precipitated high molecular weight proteins using SSA following Schop (2020), to compare the appearance of LMWN using our modified methodology on the same samples. Additionally, FL and IAPR were used at the end of the small intestine phase to simulate brush border enzyme activity, aiming to enhance LMWN production. FL is a peptidase originating from *Aspergillus oryzae*, including 2 aminopeptidases, 2 dipeptidyl peptidases, 3 endopeptidases, and 1 α-amylase (Merz et al., 2015). Intestinal acetone powder from rat (**IAPR**) is derived from the small intestine epithelium and used as a source of brush border enzymes (Kan et al., 2021). SSA cannot completely precipitate all high molecular weight proteins (Ross, 2013) and specifically targets proteins such as β-lactoglobulin while not effectively precipitating bovine serum albumin (Greenberg and Shipe, 1979). This incomplete precipitation likely resulted in some high molecular weight peptides remaining in solution among the tested feed ingredients, potentially leading to an overestimation of appearance of LMWN during the small intestine phase. Even though FL or IAPR are exopeptidases which have a larger range of cleavage sites than pancreatic enzymes including trypsin and chymotrypsin as endopeptidases (Hooton et al., 2015), their hydrolysis of high molecular weight N may have occurred within this unprecipitated fraction. Additionally, the breakdown of large soluble peptides could not be measured using the method employed in this study. Consequently, the true impact of FL and IAPR on appearance of LMWN might be obscured, making it seem as though these treatments had no effect. Besides SSA precipitation, various analytical approaches present distinct limitations: cutoff filters demonstrate operational complexity; methanol precipitation shows variability dependent on protein matrix composition; while o-phthaldialdehyde derivatization (Sousa et al., 2020, 2023) exhibits both technical complexity and amino acid dependency that complicates N balance calculations. The observed similarity of patterns of N solubilization and appearance of LMWN implies that solubilized proteins, likely exposing more peptide bonds for enzymatic cleavage (Achouri et al., 1998), are instantly degraded in smaller units. As a result, measuring the appearance of LMWN did not provide much additional information for understanding the kinetics of digestion in complement to the quantification of N solubilization.

### Nitrogen from rapeseed meal and fish meal solubilized and hydrolyzed more slowly than from barley and peas at pH 2

In general, final N solubility at the end of small intestine phase for RSM, fish meal, barley, and peas was in the range of previous in vitro findings. These prior studies indicated higher N solubility values for fish meal (90.3 ± 1.2%) and barley (84.2% to 87.4%) than RSM (84.2% to 86%) and peas (95.6%) (Eid and Matty, 1989; Boisen and Ferna, 1995; Boisen, 2007; Wilfart et al., 2008). These values are also in line with tabulated standardized ileal digestibility values provided by CVB (Centraal Veevoeder Bureau, 2021). Barley and peas, that were ground but not heat-processed prior to the in vitro incubations, showed already considerable N solubilization (41% and 46%) and appearance of LMWN (36% and 35%) at pH 4 (0 to 90 min of the stomach phase), whereas for fish meal the majority of N solubilized (52%) only at pH 2. For RSM, the N solubilization and the appearance of LMWN showed a more gradual increase over all phases compared with other feed ingredients. Rapeseed meal and fish meal are processed ingredients that underwent heat treatment (Heuzé et al., 2015; Heuzé et al., 2020). During processing, the stereochemical structure of proteins can be destroyed but also intermolecular β-sheets can be formed, which indicates protein aggregation (Salazar-Villanea et al., 2016; Xu et al., 2019). This aggregation impedes effective hydrolysis by pepsin, explaining the marginal N-solubilization for these ingredients at pH 4. Moreover, RSM and fish meal have a higher buffering capacity than barley and peas, and are more resistant to acidic conditions in water (Giger-Reverdin et al., 2002; Montañez-Valdez et al., 2013). As we used a static incubation assay where pH was only adjusted at the start of each phase, variations in buffering capacity among feed ingredients might affect the realized pH that substrates were exposed to during incubation. The isoelectric points (**pI**) of RSM, fish meal, barley, and pea proteins all ranges from 5.5 to 6.0 (Gehring et al., 2011; Mohanta et al., 2019). fish meal and RSM resist changes in pH more effectively, resulting in pH levels that deviate further from the target pH of 4 compared with barley and peas. This closer proximity to the pI of 5.7 for fish meal and RSM leads to less N solubilization than for barley and peas, potentially explaining some variation in N solubilization and appearance of LMWN observed at pH 4. However, due to the high volume of liquid relative to the incubated substrates, the effect of buffering capacity on the pH was limited.

### fish meal showed the greatest increase in nitrogen solubilization and hydrolysis at pH 2 compared with other feed ingredients

Among the selected feed ingredients, fish meal, being an animal product without fiber, showed the greatest increase in N solubilization and hydrolysis, making it more responsive to the pH decrease from 4 to 2 in the stomach phase compared with the plant-based ingredients. The plant cell wall matrix functions as a physical hindrance, encapsulating proteins and inhibiting their hydrolysis (Rovalino-Córdova et al., 2019; Holland et al., 2020), contributing to 25% and 40% lower N solubilization in 90 to 180 min of stomach phase at pH 2 for barley and peas compared with fish meal. Despite being extensively processed, rapeseed meal still contains considerable amounts of water insoluble—and poorly degradable—cell wall material (Pedersen et al., 2017). For RSM, approximately 24% of the total N is bound to the NDF-fraction (Lannuzel et al., 2022), underscoring the relative poor accessibility of RSM proteins to proteases. Zein was not included in the analysis as its N solubilization and appearance of LMWN kinetics clearly differed from that of the other feed ingredients. We visually observed zein coagulating and forming a gel, especially in the early stage of the stomach phase, in accordance with previous observations by Selling et al. (2005). Even though in the late stage of stomach phase and small intestine phase, those coagula were observed to be partly broken down, small clumps still existed, hampering representative sampling from the suspensions and resulting in considerable variation between replicate measurements especially during small intestine phase (Table S1). Moreover, gelation of zein has been observed to intensify as the pH increased from 2 to 6, with further enhancement noted as the pH transitioned from 6 to 12 (Nonthanum et al., 2013). This gelation may hinder enzyme accessibility. We observed a decrease in N solubilization and appearance of LMWN when adjusting the pH from 2 to 6.8 at the start of the intestinal phase (Table S1). The overall true ileal digestibility of AAs for zein was reported to be around 77% in pigs (van der Wielen et al., 2023), which is comparable to the N solubility and appearance of LMWN of one of the replicates in the current in vitro analysis (72% and 71%). As previously discussed, RSM, fish meal, barley, and peas all showed patterns in line with tabulated standardized ileal digestibility values. Unlike zein, these feed ingredients did not exhibit gelation and demonstrated a more gradual increase in N solubilization and appearance of LMWN throughout the stomach and small intestine phases. By comparing the behavior of zein with the other selected feed ingredients, we can better understand the reasons behind the relatively low ileal digestibility of zein.

### Added value of the modifications on the measurement of N solubilization and hydrolysis kinetics

This modified assay provides opportunities to obtain more insight in the kinetics of N solubilization and appearance of LMWN in stomach and small intestine phase. Key modifications include the consideration of pH reduction from 4 to 2 to mimic gastric digestion and the inclusion of brush border enzymes to reflect protein hydrolysis in the small intestine. These adaptations provide a more comprehensive understanding of the kinetics of N solubilization and the appearance of LMWN during digestion. The assay facilitates predictions of the transit of soluble proteins from stomach to small intestine, as well as the subsequent hydrolysis and absorption processes across various protein sources for feed evaluation purposes. In feed formulation, specific goals, such as synchronizing the supply of starch and protein or ensuring a gradual and consistent release of amino acids in the small intestine to minimize protein oxidation, need to be achieved. The detailed analysis of protein solubilization and hydrolysis kinetics provided by this assay supports the selection and optimization of alternative protein source combinations, making it a valuable tool for feed evaluation and formulation.

## Conclusion

The majority of N was solubilized and hydrolyzed for the selected feed ingredients during the stomach phase, primarily following the pH decrease from 4 to 2. fish meal N solubilized and hydrolyzed the fastest at pH 2, resulting in the highest N solubility (73%) and LMWN (68%) at the end of the stomach phase among the tested ingredients. Conversely, RSM exhibited the lowest N solubility at 46% and the lowest LMWN at 39%. These results highlight the effectiveness of the modified in vitro assay in capturing differences in N solubilization kinetics and hydrolysis behaviors, consistent with known ileal digestibility patterns. While the additional incubation with FL or IAPR did not enhance LMWN appearance, the assay provides a foundation for refining methods to more accurately measure LMWN. Moreover, the distinct behavior of zein, attributed to its gelation properties, underscores the capability of the modified in vitro assay to investigate ingredient-specific characteristics. The modified in vitro assay provides valuable insights into the solubilization and hydrolysis behavior, reflecting differences in protein digestion kinetics and ileal digestibility.

## Supplementary Material

skaf190_suppl_Supplementary_Table_S1

## Abbreviations

AA: amino acids
FL: Flavourzyme
IAPR: intestinal acetone powders from rat
LMWN: low molecular weight nitrogen compounds
N: nitrogen
RSM: rapeseed meal
TSN: total solubilized nitrogen
