# Supplementary material for: Quantifying protein digestion kinetics of feed ingredients using a modified in vitro incubation assay
Source: J Anim Sci. 2025 Jun 18;103:skaf190. doi: 10.1093/jas/skaf190 (PMC12267147; doi:10.1093/jas/skaf190)
Supplement: skaf190_suppl_Supplementary_Table_S1 [file skaf190_suppl_supplementary_table_s1.docx]

Supplement

Table 1. Nitrogen (N) solubility and appearance of low molecular weight nitrogen compounds (LMWN) (%) of total protein for zein at different time points during a three-step enzymatic *in vitro* incubation conducted in replicate representing by mean and standard deviation (SD)^1^.

|  | N solubility, % | | LMWN, % | |
| --- | --- | --- | --- | --- |
|  | mean | SD | mean | SD |
| Stomach, min | | | | |
| 0 | 4.0 | 0.3 | 3.1 | 0.1 |
| 10 | 8.5 | 0.2 | 5.0 | 0.6 |
| 20 | 12.5 | 0.0 | 9.5 | 0.3 |
| 30 | 18.3 | 0.1 | 15.4 | 0.8 |
| 60 | 31.1 | 1.1 | 29.0 | 0.4 |
| 90 | 43.0 | 1.2 | 38.2 | 1.7 |
| 100 | 56.5 | 6.2 | NA^1^ | NA^1^ |
| 120 | 68.0 | 6.0 | 62.8 | 5.5 |
| 180 | 103.8 | 10.2 | 97.5 | 9.6 |
| Stomach + Small intestine, min | | | | |
| 195 | 45.1 | 12.1 | 46.5 | 15.5 |
| 205 | 34.3 | 1.9 | 34.8 | 6.3 |
| 215 | 48.5 | 16.1 | 46.2 | 17.0 |
| 225 | 42.9 | 10.0 | 42.9 | 13.5 |
| 255 | 35.7 | 2.0 | 44.2 | 12.0 |
| 315 | 37.0 | 2.3 | 35.1 | 5.0 |
| 375 | 37.0 | 21.9 | 42.0 | 18.5 |
| 435 | 44.3 | 28.1 | 43.6 | 27.3 |

^1^*In vitro* incubation with pepsin at pH 4 for 90 min and pH 2 for 90 min (stomach) followed by incubation with pancreatin at pH 6.8 for 240 min (small intestine).

^2^Missing value.
